# Community health workers for non-communicable diseases prevention and control in Bangladesh: a qualitative study

**DOI:** 10.1186/s41256-020-00182-z

**Published:** 2020-12-24

**Authors:** Lal Rawal, Shamim Jubayer, Sohel R. Choudhury, Sheikh Mohammed Shariful Islam, Abu S. Abdullah

**Affiliations:** 1 School of Health, Medical and Applied Sciences, Central Queensland University, Sydney Campus, 400 Kent Street, Sydney, NSW Australia; 2 Physical Activity Research Group, Appleton Institute, Central Queensland University, Adelaide, SA Australia; 3 Translational Health Research Institute (THRI), Western Sydney University, Sydney, Australia; 4 National Heart Foundation Hospital and Research Institute, Mirpur 2, Dhaka, 1216 Bangladesh; 5 Institute for Physical Activity and Nutrition, Deakin University, Melbourne, Australia; 6 Global Health Research Center, Duke Kunshan University, 8 Duke Avenue, Kunshan, 215347 People’s Republic of China; 7 Duke Global Health Institute, Duke University, Durham, NC USA; 8 Boston University School of Medicine, Boston Medical Center, Boston, MA USA

**Keywords:** Community health workers, Non-communicable diseases, Health policy, Health systems, Challenges and barriers

## Abstract

**Background:**

The increasing burden of Non-Communicable Diseases (NCDs) in Bangladesh underscores the importance of strengthening primary health care systems. In this study, we examined the barriers and facilitators to engaging Community Health Workers (CHWs) for NCDs prevention and control in Bangladesh.

**Methods:**

We used multipronged approaches, including *a. Situation analyses using a literature review, key personnel and stakeholders’ consultative meetings,* and *exploratory studies*. A grounded theory approach was used for qualitative data collection from health facilities across three districts in Bangladesh. We conducted in-depth interviews with CHWs (Health Inspector; Community Health Care Provider; Health Assistant and Health Supervisor) (*n* = 4); key informant interviews with central level health policymakers/ managers (*n* = 15) and focus group discussions with CHWs (4 FGDs; total *n* = 29). Participants in a stakeholder consultative meeting included members from the government (*n* = 4), non-government organisations (*n* = 2), private sector (*n* = 1) and universities (*n* = 2). Coding of the qualitative data and identification of themes from the transcripts were carried out and thematic approach was used for data analyses.

**Results:**

The CHWs in Bangladesh deliver a wide range of public health programs. They also provide several NCDs specific services, including screening, provisional diagnosis, and health education and counselling for common NCDs, dispensing basic medications, and referral to relevant health facilities. These services are being delivered from the sub-district health facility, community clinics and urban health clinics. The participants identified key challenges and barriers, which include lack of NCD specific guidelines, inadequate training, excessive workload, inadequate systems-level support, and lack of logistics supplies and drugs. Yet, the facilitating factors to engaging CHWs included government commitment and program priority, development of NCD related policies and strategies, establishment of NCD corners, community support systems, social recognition of health care staff and their motivation.

**Conclusion:**

Engaging CHWs has been a key driver to NCDs services delivery in Bangladesh. However, there is a need for building capacity of CHWs, maximizing CHWs engagement to NCD services delivery, facilitating systems-level support and strengthening partnerships with non-state sectors would be effective in prevention and control efforts of NCDs in Bangladesh.

**Supplementary Information:**

The online version contains supplementary material available at 10.1186/s41256-020-00182-z.

## Background

Globally, the Non-Communicable Diseases (NCDs) have become the leading cause of death and disability and are the major ongoing public health problems [1–3]. In 2016, of the 57 million death occurred worldwide, 71% was due to NCDs [4]. This burden is alarming in low- and middle-income countries (LMICs), where over 85% of premature deaths and 78% of all NCD deaths occur [5]. The major NCDs responsible for these deaths and disability are cardiovascular diseases (CVD) (44% of all NCD deaths); cancers (22%); chronic obstructive respiratory diseases (9%); and diabetes (4%) [4].

Like other LMICs, Bangladesh is experiencing rapid demographic and epidemiological transitions [6–8], and subsequent rise in ageing population and the burden of NCDs [7–9]. The Global Burden of Disease Study estimated that the proportion of deaths due to NCDs in Bangladesh increased from 43.4% in 2000 to 60% in 2010 and 66.9% in 2015 [10, 11]. The rapid increase of NCDs mortality and morbidity in Bangladesh has presented a major threat to Bangladesh’s existing healthcare systems, which are mainly geared towards addressing communicable diseases [12, 13]. This also affects overall socioeconomic development of the country, which poses significant threat to achieving Sustainable Development Goals by 2030 [14]. The impact of NCDs on the national economy, communities, families and individuals is unbearable [15–17], and this is likely to be more serious in coming years [18–20]. Recent studies have shown that NCD risk factors such as overweight, underweight, hypertension, dyslipidemia, physical inactivity, tobacco smoking and low consumption of vegetables were common among adults living in urban [21, 22] as well as rural areas [23, 24] including adults of all economic quintiles [15].

In recent years, the government of Bangladesh has taken several initiatives to combat NCDs [25, 26]. National NCD action plan has been developed, a dedicated non-communicable disease control (NCDC) programme has been established at the Directorate General of Health Services, within the Ministry of Health and Family Welfare. In order to provide the NCD services in the doorstep, one of the key initiatives government has taken since 2012 is, establishing NCD corner in Upazila health complexes (UHCs) levels. These NCD corners, in parallel with the existing services in UHCs, provide NCD and related services. Each NCD corner is expected to have a dedicated team and necessary equipment [12]. However, the NCD corners face shortages of trained health care providers, lack of necessary supplies and logistics, lack of basic medical equipment and medication, and the problem of proper recording and reporting of NCD services being delivered [27].

The Community Health Workers (CHWs) in Bangladesh are those health cadres working in public sector health facilities including Community Health Care Provider (CHCP), Assistant Health Inspector (AHI), Health Inspector (HI) and Family Welfare Assistant (FWA). These CHWs receive some form of formal training. The duration ranged from 12 weeks for CHCP to over three years for HI [28]. The services provided by these CHWs varied according to their category and includes: (i) basic services and health education for maternal and child health; (ii) integrated management of childhood illness (IMCI); (iii) reproductive health and family planning services; (iv) expanded program on immunization (EPI); (v) nutritional education and micro-nutrient supplements; (vi) health education & counselling; and (vii) treatment of minor ailments, common diseases & first aid. In recent years, the CHWs in some areas have also started providing services for NCDs, especially random blood glucose test for those at risk of developing diabetes and provide health education and counselling services. However, there is no proper referral and follow up mechanism from one health facility to another. It is up to the patient where to go for further management or follow up.

While Bangladesh is committed to offering universal access to health services for NCDs, no options to supplement the existing primary healthcare delivery system has been identified. Information on the training of CHWs, the process to deliver healthcare interventions through CHWs, and the effectiveness of CHW-led intervention are lacking. Identification of the facilitators and barriers to CHW-led implementation of NCD related services can provide policymakers and practitioners with a portfolio of options to address the growing demands for NCD-related healthcare services. Therefore, our study aimed to explore the barriers and facilitators of engaging CHWs in delivering NCD related services in Bangladesh.

## Methods

### Study design, site, and participants

We used a grounded theory approach [29, 30] involving key informant interviews (KIIs) with policymakers, in-depth interviews (IDIs) and focus group discussions (FGDs) with CHWs, and stakeholders’ consultative meetings with key stakeholder members. This study was conducted in both urban and rural areas of three districts (Sylhet, Chandpur and Dhaka) in Bangladesh. Of four FGDs, two were conducted in rural and two in urban areas. FGDs were held in the community clinics of rural areas and urban primary health care centres of urban areas during the months of December 2016 and May 2017.

Similarly, the four IDIs (2 in urban and 2 in rural areas) with CHWs were conducted. The FGDs and IDIs were focused on exploring the perceptions of CHWs on NCD, their current workload, capacity in delivering NCD focused preventative care, and the challenges faced by them in the community. The KIIs were conducted in the natural setting of experts (i.e. workplace environment), and these participants were selected based on their current involvement and responsibilities with primary health care service delivery, NCD surveillance, and research background. A stakeholder meeting was organized by the National Heart Foundation Hospital and Research Institute on 31st December 2016 to obtain the views of government and non-government stakeholders on the role of community health workers in combating NCD in Bangladesh.

### Study tools development

This study was a part of the multi-country study of Asia and Pacific regions [28], where we adopted the study tools developed by the multi-country project team to Bangladesh’s context. The qualitative interview guides were guided by the extensive review of relevant literature, government reports and guidelines, available relevant tools, and consultation with government and non-government officials. In order to check face validity, the developed interviewed guide was reviewed by the experts working in the field of NCDs. The interview guide was translated into the Bengali language by a bilingual professional transcriber and was pre-tested in the study setting to ensure cultural and contextual appropriateness to the Bangladesh context.

### Data collection

Purposive sampling was used to choose the participants for this study, and study participants were recruited from three districts. Three main approaches were used for data collection (i) One stakeholders meeting with senior government and non-government officials, researchers, academician and clinicians (*n* = 9) (ii) four FGDs (2 FGDs in rural and 2 FGDs in urban areas, *n* = 29) with the community clinic service providers and (iii) KII with experts representing the government and non-governmental organizations (*n* = 15). See Table 1 for details. Data were collected during December 2016 and May 2017. All interviews were conducted by two trained research officers (ROs) from the National Heart Foundation Hospital and Research Institute, a national research-based organization in Bangladesh. All interviews and discussions were conducted in local Bengali language and were audio-recorded; one RA led the discussion while another RA took notes during fieldwork. ROs examined the quality of the initial transcriptions with the audio records to confirm the transcribed content was consistent with the participants’ voices. The country principal investigator (SC) checked the final transcriptions. After finishing all the quality checks of transcription, the translation was conducted by a research officer. The initial translation was delivered by the local transcript translator, upon a written agreement on confidentiality. Then, the revised translation was jointly done by the research officer, research fellow, and the PI. The three members conducted regular discussions and reviews to ensure the quality of information and the content fit in the context, with adjustment made to the vocabularies that caused discrepancies and misapprehension.

**Table 1:** Approaches used for qualitative data collection

Data collection types	Number of participants
In-depth qualitative interviews	4 IDIs with MO, CHCPs /CHWs
Key informant interviews	15 KIIs with central level policy makers and health managers
Focus group discussion	4 FGDs with Community health workers and 2 FGDs with Urban health workers s, *n* = 29
Stakeholder consultative meetings	One at the beginning of the study with participants from government (*n* = 3), NGOs (*n* = 5), private sector (*n* = 1) and universities (*n* = 2)

### Data processing and analysis

The audio files of the collected data were transcribed verbatim by one of the investigators of the research team (SJ). Two of the team members went through all the transcripts independently, and then coding was done. We adopted an inductive approach in the coding process where two members (SJ and LR) of the researcher team identified the codes by comparing and contrasting the meanings and patterns as expressed by study participants. The identified codes were further checked by another two researchers (SC and AA) for accuracy. All team members reviewed the codes and then finalized the emerging themes from the data. The coding of the transcripts and the identification of emerging themes were carried out using a thematic approach, as recommended by Nowell et al., (2017). Four major themes identified include: (i) Health care delivery around *NCDs in Bangladesh; (ii) General public health programs delivered by CHWs (iii) NCD-specific programs provided by CHWs; and (iv) Facilitating and enabling factors in engaging CHWs in NCD control & prevention.* In order to maintain confidentiality, all the data were de-identified and anonymized throughout the transcription and interpretation of the findings.

The collection and analysis of data from qualitative interviews adhered to the Standards for Reporting Qualitative Research (SRQR) [31] and strategies were employed to enhance the trustworthiness (credibility, transferability, dependability, confirmability and transferability) of the study findings [32, 33]. This included checking the data for accuracy, organising debriefings for completeness of data (LR, and SJ). See Additional file 1, SRQR Checklist as Supplementary document.

## Results

### Participants’ characteristics

The details of the participants’ characteristics are presented in Table 2. Of total 14 health facilities included in this study, five were from the urban setting, six from rural and three from the central level health facilities.

**Table 2:** General characteristics of the FGD participants (*n* = 29), IDI participants (*n* = 4) and KII participants (*n* = 15) of Bangladeh COACH study

Characteristics	FGDs (FGDs; Total participants, *N* = 29)	IDIs (*N* = 4)	KIIs (*N* = 15)
*Work setting*			
Rural health centres	15	2	
Urban health centres	14	2	
Central level health facility/policy level			15
*Age by category*			
20–29	4		
30–39	29	2	
40–49	4	2	4
> =50	6		11
*Gender*			
Male	17	3	3
Female	12	1	2
*Duration of job in the public health facilities*			
< 10 years	5	4	
10–19 years	18		4
20–29 years	6		11

#### NCDs related health services delivered by CHWs in Bangladesh

Community Clinics (CCs) are the grassroots level public sector health facilities in Bangladesh. The services are provided by the Community Health Workers, including CHCP along with FWA and HA. The common NCD and related services provided by these CHWs include a screening of common NCDs such as diabetes and hypertension. However, the services for the detection of other NCDs such as COPD and cancer are not yet available. Basic medication for diabetes and hypertension is also available at the community clinic level. However, for further investigation and confirmatory diagnosis of NCDs and appropriate management, the patients are referred to upper-level health facilities, such as Upazila health complex (sub-district level health facility) or district level health facility, where specialized health services are available. The FGD members reflected this opinion during the FGD.

*“When the patients with the possible non-communicable disease come to our community clinic, we tell them about the risk factors of NCDs, but we realize more awareness and health education needs to motivate the community. We offer BP measurement, Blood sugar test using a glucometer, minor treatment of RTA and some basic biochemical test.”* – *IDI 1-Medical Officer, Urban Primary Healthcare Centre, Dhaka*

Notably, CHWs said that they usually provide necessary advice and counselling for common NCDs to the people who come to see them. The CHWs provide advice related to dietary habits, the importance of physical activity, health education and healthy behaviour to prevent NCDs when anyone come to consult them. Most of the CCs have limited laboratory facilities available, and the CHWs diagnose hypertension but no other NCDs because of lack of resources and training. Some of those CCs, which have glucometers available to test blood sugar, are most of the time, out of order or the supplies such as glucometer strips or batteries ran out. One participant said:

“*BP machine and glucometer, along with other measuring tools, are available in our CCs. But the problem is we don’t have enough strips here at the CCs, right now because of the shortages of glucometer strips, so I can’t check the blood glucose level using the glucometer.*” – *FGD1-Health Assistant-Community Clinic, Sylhet district*

In general, CHCPs received 12-weeks basic training, mostly focused on maternal and child health and HCPs and AHI/HI received one-day training on NCD prevention and management. The NCD training included a general screening of NCDs, health education, counselling and basic care for common NCDs. One CHCP expressed:

*“We don’t have any extensive training on the management of common NCDs, i.e. diabetes, hypertension but these are the very common finding in our regular activity and identified patient wants to get their treatment at the community level”* - *IDI2 Health Supervisor, Urban PH Clinic, Lalbagh, Dhaka*

To date, there is no systematic approach in place for a referral and follow up of a patient. These are up to the patient to choose the health facility they want to go for further investigation and management. Suspected patients are referred to UHCs for further investigation, diagnosis and necessary management of NCDs.

To provide medication, the NCD related drugs are supplied within the regular drug supply schedule, which in general takes longer time, even up to four months once the requisition is made. Also, the uncertainties exist in terms of discrepancy between requisition and actual supply. One of the KIIs shared that:

*“to date, over 29 different types of medicines are supplied to community clinics from the government. They are mostly antibiotic, vitamins, eye ointments, salbutamol, anti-helminths, oral saline and acetaminophen product. These drugs also have more demand for community people. The anti-hypertensive and diabetic drug is not yet available at community clinic though community people are regularly demanding supply for those from CHCP”. KII1 Line Director, (NCD), Director General of Health Service*

In terms of recording and reporting, participants noted that there is no separate registration for NCDs conditions or related risk factors. The information regarding the patients with NCDs conditions is recorded and reported along with patients of other health conditions.

#### Barriers and challenges to engaging CHWs in the NCD prevention and control services

This study identified several challenges and obstacles to engaging CHWs for NCD prevention and control in Bangladesh. The challenges are mainly related to workload, inadequate training, poor system-level support, inadequate remuneration, delivery of services and the availability of resources. The challenging factors are structured in the following key sub-themes:

##### Lack of trained human resources for health

At present, the CHWs job description primarily involves providing services for communicable diseases, MNCH care, nutrition, immunization and health education and counselling services. Participants opined that, to date, no clear job description had been given, which would involve NCD services too. However, in recent years, the health facilities have initiated providing essential preventive and care services for those with NCDs conditions. The CHWs are currently multi-tasking and responsible for both the administrative and technical tasks. One KII opined that:

*“Our jobs have so far given mainly focused on providing services for communicable, maternal and child health and related services. We still do not have a clear job description on NCD services, however, we know somehow about preventive aspects and also basic NCD services, such as medication for diabetes and hypertension”- KII2, Line Director, Community Based Health Care, Director General of Health Service*

Further, the CHWs are not yet trained on NCDs screening and providing basic NCD care which hinders them providing NCD services. This may require providing focused training on NCDs and making necessary supplies (i.e. medical equipment and medicines) available. Inadequate number of health workers at the health facilities often resulted in increased workload on the existing staffs, thus affecting the effective health services delivery, including NCDs services. One health worker expressed that:

“*There are no trained CHCPs, AHI/HI at this CC. Even still now, I have not received any training on NCD. We all received maternal and child health-related training.* - *FGD2, Community Clinic, Uttar Matlob, Chandpur*

Another participant also added that there had been a shortage of health care providers too:

*“ … . but the problem is the staffs need to be involved with their regular activities, and we have staff shortage, especially paramedics. So due to support staff shortage, there is no one to support the dedicatedly NCD patients at the Community Clinic.*” – *KII3, Principal Scientific Officer, Institute of Epidemiology Disease Control and Research, Director General of Health Service*

##### Lack of regular supply of logistics and medicine

CHWs reported frequent delays in the delivery of medication supplies for common NCDs (i.e. hypertension and diabetes) at the community clinic from the sub-district hospitals. This has hampered their service delivery to the community members and raised dissatisfaction among the public. One participant opined:

“*Yes, we have received glucometer, BP machine, weight and height machine for the CC. But now the problem is, there is a shortage of battery and strips for the glucometer for which we can’t use the glucometers.*” - * FGD3 Health inspector, Rakhal Gonj Community Clinic, South Surma, Sylhet*

Another participant in the FGD expressed that, once a year, the CCs provide a requisition for medicine. The medicine comes at the CS office, and from where the CCs take what is being allotted for them. The NCD related medicines come with the regular medicine supply, but the supply schedule is uncertain. Also, the amount and types of medicine sent for treating NCD patient is not adequate.

*“For the outpatients, the type of medicines dispensed depends on the available medicines and supply. We always try to provide whatever medicines are available at the CCs. But for the inpatients, we have medicines required to treat the NCD patients at Upazila level.” – KII4 Upazilla Health and Family Planning Officer South Surma, Sylhet.*

Further, the health facilities, in particularly the CCs, are facing other logistics-related challenges, such as shortages of adequate space, lack of furniture, and inadequate necessary instruments to deliver NCD related services. One FGD participant said:

“*The CCs are facing many problems. There is a lack of proper instrumental support. At my CC, we received only a BP measurement set. We are waiting for other equipment to arrive. We have no glucometer for screening NCD cases as most of the required blood tests we now advised them to go UHC, but we need available immediate screening and measurement tools.*” – *FGD4 Zohirbad Nadamodi Community Clinic Uttar Matlab, Chandpur*

Coupled with above-described challenges, the participants also identified other key challenges including: inadequate health systems priority and lack of standard operating procedure to NCDs prevention and care, no specific policy documents prioritizing NCDs care and services as core activities of the health facilities, and lack of awareness on NCDs risk factors, NCDs symptoms and delay in seeking care by the patients at the community level.

#### Facilitating and enabling factors in engaging CHWs in NCD prevention and control

Participants identified following facilitating factors to support engagement of CHWs in NCDs prevention and control.

##### Supportive policy initiatives to NCDs prevention and control

Over the past several years, the government of Bangladesh has initiated several NCD prevention and management related initiatives. Policies and guidelines to NCDs prevention and control at the systems level have been developed, an NCD control program at the DG health services has been created - to primarily looking after the NCD issues. To this end, the NCD corners at the sub-district level health facilities (UHCs) has been introduced, and several efforts to increase NCDs prevention and control measures at the community level have been initiated. One participant opined that:

*“The government of Bangladesh has given priority for NCDs prevention and control. The government also has established over 300 NCD corners in the country, and we have been able to somehow implement NCD services through these NCD corners in the country” – KII5 Divisional Director, Sylhet Division, Director General of Health Service.*

Further, in recent years few necessary NCD drugs have been added into the essential drug list and made these essential services drugs (ESDs) freely available at the community health facilities.

##### Recruitment and fulfilling the vacant positions in health facilities

The CHWs believed that the initiatives taken by the Directorate General of Health Services, Bangladesh, to fulfil the vacant positions in the health facilities will ensure delivery of quality health services in the community level, including NCDs services. They also thought that the retention of health workers is another important aspect of delivering NCD and related services. This is one of the keys facilitating factors the CHWs identified in this study. One participant said:

*“Government of Bangladesh has taken a good initiative to recruit new positions and fulfil the vacant positions in all health facilities. Retention of health workers to the assigned health facility, in particular, the rural areas is equally important as recruitment and deployment” – KII6, Head of the Department of Epidemiology, National Institute of Preventive and Social Medicine.*

##### Job satisfaction

Despite several challenges the CHWs are facing, they shared that they have a good level of personal satisfaction to the services they are providing. CHWs said that serving people in the community reciprocate the respect and recognition from the community members. One of the participants shared:

*“I feel great that I have got the opportunity to work at the health facility in my community, and I am serving people in my area. When people tell me that I have done a good job, I feel very proud and happy, and that makes me more responsible” – IDI-4, CHCP, Rakhal Gonj Community Clinic, South Surma, Sylhet.*

##### CHWs as key drivers to NCDs prevention and control

Most of the KIIs, IDIs and the participants of the stakeholder consultation meeting viewed that the CHWs have been playing a vital role for NCDs prevention and management services. One KII emphasized:

*“Traditionally, the CHWs have been trained and given tasks to deliver communicable diseases and related services. However, the situation in Bangladesh has changed where we are dealing with NCDs rather than communicable diseases. The CHWs have been well recognized to play vital roles in the prevention and management of NCDs. Of course, the CHWs need a range of policy and systems support and guidance, which they have been provided by the government of Bangladesh. KII-7, Manager, Directorate General of Drug Administration*

##### Collective efforts to NCDs prevention and control

The policy level participants (health managers, researchers, academicians and clinicians) stated that the Ministry of Health and Family Welfare, Bangladesh has given priority to NCDs prevention and control in Bangladesh. There has been a government level commitment. However, there is a need for working together and collaborating with other ministries and non-state sector to optimize the efforts for NCDs prevention and control in Bangladesh. Participants also shared that the non-state sector, in particular, the NGOs and private sector have also taken several initiatives. They also provide NCDs prevention and care services, including screening, early detection, diagnosis, treatment, referral, and health education and counselling services in Bangladesh. One participant highlighted:

*“The government of Bangladesh have developed a range of NCD related policies and guidelines. We have a dedicated department (housed within the DG Health services) to look after NCD related programs in the country. However, there is a need for working together with other ministries, NGOs and private sector” KII-1, Line Director, Community Based Health Care, Director General of Health Service*

## Discussion

Like many other LMICs, Bangladesh has been experiencing the growing burden of NCDs, posing significant threats to the existing weak health care systems in the country [8–10]. In this context, our study is the first of its’ kind to explore barriers and facilitators to CHWs potential engagement in delivering NCD related services in the country. Our findings determined the role and extent to which CHWs could be engaged and what is required to support CHWs in the prevention and control efforts of NCDs in Bangladesh.

In recent years, the government of Bangladesh has taken several initiatives to combat NCDs at systems, institutional and community levels [25, 26]. To provide the NCD services in the doorstep, one of the critical initiatives government has taken since 2012 is, establishing NCD corner in UHCs levels. Findings of this study suggest that the CHWs in Bangladesh are the primary drivers of delivering preventive health and NCDs related services at the community level, which is consistent with the initiatives considered by other neighbouring countries of Asia [28, 34, 35]. Evidence shows that engaging CHWs for delivery of health services and health promotion interventions is increasing in LMICs and proven to be effective [36–39]. CHWs provide opportunities for effective implementation of health promotion interventions within the existing healthcare systems without engaging new personnel with whom families have had no prior contact. Several countries in the South and South-East Asian regions (i.e. Bangladesh, China, Nepal, and Vietnam) have delivered different public health programs through CHWs; and the effectiveness of these programs are reported [40–42]. However, scaling up of these programs and integrating these within the existing healthcare delivery systems are important to deliver ongoing general health as well as NCD related services in Bangladesh. While the ongoing problem of COVID-19 is increasing, the roles of CHWs have been even crucial to delivering regular health services as well as COVID-19 preventive and management care [43, 44]. Available evidence shows that the people with chronic conditions are highly affected by COVID-19 (high mortality) than the general population, hence, the role of CHWs to effectively provide NCD care during the ongoing COVID-19 pandemic has critical public health importance [45].

In this study, CHWs were highly interested in delivering NCD related services in their community. CHW’s motivation in healthcare delivery can be taken as a key factor in implementing NCD related services and achieving Sustainable Development Goal 3, “Health and Wellbeing” in Bangladesh by 2030 [14]. Results from our study are in concordance with the findings from other settings where CHWs expressed the need of capacity building for the prevention and control of NCDs [27, 39, 46]. These findings are plausible because in past decades the CHWs were very much capacitated on maternal and child health services and were mostly dealing with communicable diseases. To address the shortfall of skilled CHWs and accessibility to health services for combating the growing demands for NCDs services at the peripheral level health facilities, [27, 47] measures to build the capacity of CHWs on NCDs control and consider them as members of the primary care team, in order to retain them as a committed workforce, is needed.

The current healthcare system of Bangladesh is not yet well prepared to mitigate the growing demand of NCDs. A number of key barriers both at systems and service delivery levels were identified. These include (i) lack of appropriate NCD policy guidelines, (ii) insufficient trained human resources, (iii) inadequate logistics, supplies and medications, (iv) lack of laboratory facilities, (v) inefficient referral mechanisms, and (vi) unavailability of systematic recording and reporting systems. These findings corroborate well to the ones reported in the recent studies from Bangladesh [13, 20, 27] and Vietnam [34]. Studies in Bangladesh have reported that, despite the introduction of NCD corners in the country and implementation of NCD related services, the public sector role has been limited to providing NCDs and related services to combat the growing burden of NCDs [13, 20, 25]. However, developing a functional team and a service delivery system in a resource-poor setting like Bangladesh is possible and has been highlighted by several studies, but there is a need for translating these potentials to actions [28, 48, 49].

Strengthening primary health care for tackling the burgeoning burden of NCDs in Bangladesh is critically important, where the CHWs who are based at community clinics, union-level health centres and UHCs could play a pivotal role in screening, provisional diagnosis, early management, referral and follow of patients with NCDs [28]. To achieve the universal health coverage and ensure the country meets NCD related SDGs targets by 2030 [14], Bangladesh would need re-structuring of the current healthcare delivery systems to address healthcare workforce disparities that exist in rural and remote regions [50]. Also, the country would need to take a multi-faceted approach and collaboration among professions and institutions that have traditionally used a vertical approach. Strengthening the partnership with the non-state sector would be essential to improving care and delivery of NCD related services in the country. Evidence shows that CHWs involvement in the delivery of primary healthcare can potentially result in cost and time savings without compromising the quality of care or health outcomes for patients [40–42]. Further, building capacity of CHWs with new and updated skill sets to NCDs; providing them with disease specific training focused on guidelines and protocols for screening and management of NCDs; and where possible, provide CHWs with training opportunities for prescribing all necessary list of NCD related medications, in consultation with medical doctors and specialists [51]. Revitalizing primary health care systems in the country would ensure strengthening model of NCD care both in urban as well as rural parts of Bangladesh. This would ensure building capacity of CHWs, access to and utilization of NCD services at primary care level, supply of logistics and medications, collaborations with other stakeholders, and ensure availability of trained human resources at the community level. Effective engagement of CHWs would also improve and ensure patient centred care which would then focus on dimensions of patient centred care including CHWs and patients’ relationship, availability of low-cost and affordable services, acceptability of services, and also ensure that the services are contextual and appropriate to the populations’ needs [52]. Further, adequate financing to NCDs sector is a key dimension to consider while efforts are being made to improve primary health care systems for prevention and control of NCDs [53, 54].

Our study has some limitations. First, we were unable to include participants from more than three sub-districts. Having data available from additional sub-districts and district level could have added extra insights to ensure more generalizable findings. Second, we were unable to collect data from the patients or community members, which could have added insights from the service recipients’ perspective but was out of the scope of this study.

## Conclusion

We conclude that there is a potential for using CHWs for NCDs prevention and control in Bangladesh. Several barriers and facilitators for CHWs’ potential engagement to deliver NCD related services have been identified. Our results offer suggested options to policymakers and researchers for integrating CHWs in the delivery of community-based interventions for NCDs prevention and control in Bangladesh. Effective integration of CHWs along with policy support for capacity buildings of CHWs, evidence-based practice and referrals and offer flexibility to adapt programs to the local context, are essential to delivering NCD related programs in the country. Further, we recommend developing and testing model interventions to train CHWs and to provide CHW’s-led NCD prevention and control programs in Bangladesh.

## Supplementary Information

**Additional file 1.**

## Data Availability

All relevant data are presented in this paper. Additional data could be available upon request to the corresponding author.
